# Developmental trajectories of body mass index from childhood into late adolescence and subsequent late adolescence–young adulthood cardiometabolic risk markers

**DOI:** 10.1186/s12933-019-0813-5

**Published:** 2019-01-19

**Authors:** Kolade Oluwagbemigun, Anette E. Buyken, Ute Alexy, Matthias Schmid, Christian Herder, Ute Nöthlings

**Affiliations:** 1 Nutritional Epidemiology, DONALD Study, Department of Nutrition and Food Sciences, Rheinische Friedrich-Wilhelms-University Bonn, Bonn, Germany; 2 Institute of Nutrition, Consumption and Health, Faculty of Natural Sciences, University Paderborn, Paderborn, Germany; 3 Department of Medical Biometry, Informatics and Epidemiology, University Hospital Bonn, Rheinische Friedrich-Wilhelms-University Bonn, Bonn, Germany; 4 Institute for Clinical Diabetology, German Diabetes Center, Leibniz Center for Diabetes Research at Heinrich Heine University Düsseldorf, Düsseldorf, Germany; 5 German Center for Diabetes Research (DZD), Partner Düsseldorf, Germany; 6 Medical Faculty, Heinrich Heine University Düsseldorf, Düsseldorf, Germany

**Keywords:** Body mass index, Trajectory, Latent (class) growth models, Maternal prepregnancy body mass index, Cardiometabolic risk markers, Diastolic blood pressure, High-density lipoprotein cholesterol, IL-6, IL-18

## Abstract

**Background:**

Reports on body mass index (BMI) trajectories from childhood into late adolescence, their determinants, and subsequent cardiometabolic risk markers, particularly among European populations have been few. Moreover, sex-specific investigation is necessary considering the sex difference in BMI, and the sex-specific association between BMI and some cardiometabolic risk markers.

**Methods:**

Using a sample from the DOrtmund Nutritional and Anthropometric Longitudinally Designed study, we explored sex-specific trajectories of the BMI standard deviation score (SDS) from 4 to 18 years of age in 354 males and 335 females by latent (class) growth models. The determinants of trajectory were assessed by logistic regression. We identified cardiometabolic risk markers that were highly associated with BMI SDS trajectory by random forest regression, and finally we used generalized linear models to investigate differences in the identified cardiometabolic risk markers between pairs of trajectories.

**Results:**

We observed four: ‘low-normal weight’, ‘mid-normal weight’, ‘high-normal weight’, and ‘overweight’, and three: ‘‘low-normal weight’, ‘mid-normal weight’, and ‘high-normal weight’ trajectories in males and females, respectively. Higher maternal prepregnancy BMI was associated with the ‘overweight’ trajectory, and with ‘high-normal weight’ trajectory in both sexes. In addition, employed mothers and first-born status were associated with ‘high-normal weight’ trajectory in females. BMI SDS trajectory was associated with high-density lipoprotein-cholesterol and interleukin-18 (IL-18) in males, and diastolic blood pressure and interleukin-6 (IL-6) in females. However, only males following the ‘overweight’ trajectory had significantly higher IL-18 when compared to their ‘low-normal weight’ counterpart.

**Conclusions:**

We identified sex-specific distinct trajectories of BMI SDS from childhood into late adolescence, higher maternal prepregnancy BMI as a common determinant of the ‘high-normal weight’ and ‘overweight’ trajectories, and ‘overweight’ trajectory being associated with elevated IL-18 in late adolescence–young adulthood. This study emphasizes the role of maternal prepregnancy BMI in overweight, and highlights IL-18 as a cardiometabolic signature of overweight across life.

**Electronic supplementary material:**

The online version of this article (10.1186/s12933-019-0813-5) contains supplementary material, which is available to authorized users.

## Background

Overweight (including obesity), a condition of excessive body fat accumulation, is still a major health challenge in modern societies. Individuals transiting from late adolescence into young adulthood are at a significant risk of being overweight [1]. It is widely acknowledged that overweight status in late adolescence–young adulthood extends back into childhood and adolescence [1–3]. Ample evidence from different populations demonstrated that childhood and adolescence overweight status indeed predict adult overweight status [4–8]. These findings confirm that while overweight in young adulthood is important, the age of onset and duration should not be ignored. These three dimensions–intensity, age of onset, and duration are usually captured through developmental patterns (trajectories) [9]. Therefore, it is important to identify groups of individuals following similar trajectories, and a commonly used approach is the latent (class) growth models (LCGM) [10]. Body mass index (BMI) is correlated with body fat, and it is valuable in children and adolescents [11]. While many studies have explored group-based BMI trajectories from childhood into late adolescence–young adulthood [9, 12–20], only two studies [9, 16] are among European populations. Moreover, most of these studies have few measurement occasions, a factor that negatively affects the accuracy of trajectories [21].

Exploring the trajectories of BMI enhances identification of their determinants and their association with aspects of health later on in life [3]. Identification of determinants allows anticipation of individuals at risk for high BMI trajectories, and would provide the basis on which effective intervention that could change these trajectories are implemented. High BMI is associated with abnormalities in cardiometabolic risk markers such as blood pressure (BP), high-density lipoprotein-cholesterol (HDL-c), triglycerides, fasting plasma glucose (FPG), and adipokines and other circulating proinflammatory cytokines [22, 23]. Overweight-related cardiometabolic risks are already evident in late adolescents–young adults [24].

Associations between high BMI at a single point of measurement in childhood or adolescence and abnormal cardiometabolic risk markers in late adolescence–young adulthood are well-documented [25–41]. However, only a few studies have examined associations between trajectories of BMI and cardiometabolic risk markers in late adolescence–young adulthood [9, 12, 16, 19, 20]. These studies observed associations between high BMI trajectories and high BP [9, 12, 16, 19, 20], reduced HDL-c [16], elevated triglycerides [12, 16], and insulin resistance [12, 16]. Admittedly, only a limited number of cardiometabolic risk markers have been examined. Therefore, it would be necessary to consider a broader range of cardiometabolic risk markers. Importantly, it is difficult to conclude from the above evidence that high BMI trajectory confers additional risk beyond age-specific high BMI. Intuitively, a clear distinction between these two might reveal trajectory-specific cardiometabolic risk markers, and unravel previously unappreciated links between adiposity and cardiometabolic risk.

There is a sex difference in body composition beginning in childhood and continuing into adolescence [42], a sex gap in overweight increasing with age, especially in adolescence [43], a significant interaction between trajectories and sex for some cardiometabolic risk markers [16], and sex difference in the association between BMI and cardiovascular outcomes in adulthood [44]. Moreover, prior research indicates that the relation between BMI and cardiometabolic risk markers in late adolescence may be sex-specific [45]. This evidence suggests investigations into sex-differential trajectory and sex-specific trajectory–cardiometabolic risk markers association. In addition, early life factors predict cardiometabolic risk markers later in life [46], and late adolescents–young adults often defer or discount healthy lifestyles [47]. Thus, it would be necessary to determine whether the relation between trajectory and cardiometabolic risk markers are independent of these factors.

Using annual anthropometric measurements from childhood into late adolescents from a German cohort, this study sought to: (1) identify and describe sex-specific BMI trajectories using LCGM; (2) identify baseline determinants of these trajectories; (3) investigate whether trajectory describes the relationship between BMI and certain cardiometabolic risk markers in late adolescence–young adulthood beyond BMI at specific ages; and (4) given the findings of aim 3, to investigate whether individuals following different trajectories also have significantly different level of cardiometabolic risk markers, that is independent of baseline determinants and late adolescence–young adulthood lifestyle factors.

## Methods

### Study design

The DOrtmund Nutritional and Anthropometric Longitudinally Designed (DONALD) study is a longitudinal (open cohort) study, collecting detailed data on diet, growth, development, and metabolism between infancy and adulthood [48]. This study uses a convenient sampling scheme; hence, children from high socio-economic families are over-represented [48]. Experienced nurses conducted anthropometric measurements throughout the follow-up period, which include annual measurements of height and body weight. Informed written consent was obtained from parents and from participants themselves on reaching adolescence. The Ethics Committee of the University of Bonn, Germany approved the study.

### Study participants

The current study includes participants who were singletons, full term (36 to 42 weeks) and birth weight of ≥ 2500 g. For the present analysis, we used anthropometric measurements from age 4 to 18 years. Trajectory analysis requires at least three measurements [49], therefore we considered individuals with at least one measurement in childhood (4–9.9 years), early adolescence (10–14.9 years), and late adolescence (15–18 years).

### Variable assessment

#### Baseline parameters

Baseline parameters such as gestational characteristics and birth anthropometrics from was retrieved maternal gestational record. These include sex, birth weight (g) and length (cm), birth order (from first to fifth), maternal prepregnancy BMI (MppBMI, kilograms/meters squared), gestational weight gain (GWG, kilograms). Breastfeeding duration (weeks), and family and socioeconomic characteristics around birth were obtained from maternal interview. Family and socioeconomic characteristics around birth include smoking household (non–smoking household, smoking in household (numbers of smokers), maternal education (according to the German education system: “Abitur”/“Fachhochschulreife” (technical school/high school), “Realschulabschluss” (secondary school), and “Hauptschulabschluss” (primary school), maternal employment (full-time and part-time, (early) retiree, unemployed, housewife, vocational trainee (including students), temporary leave, and maternal leave).

#### Dietary intake and lifestyle factors in late adolescence–young adulthood (18–39 years)

Three-day weighed dietary records assessed dietary intake. We calculated individual means of daily nutrient intake from these records, using our continuously updated in-house food composition database. In addition, participants reported alcohol consumption (drinker or non-drinker) and smoking status (non-smokers or smokers-daily, once a week, several times in a week, seldom).

#### Cardiometabolic risk markers in late adolescence–young adulthood

Participants underwent a medical examination that included measurement of systolic blood pressure (SBP) and diastolic blood pressure (DBP), and obtaining a fasting blood sampling [50]. We retrieved, processed, and measured more cardiometabolic risk markers from fasting blood samples of participants who fulfilled the inclusion in the trajectory analysis. Serum cholesterol (total, LDL-C and HDL-C) were measured at the Paediatric Clinic Dortmund with the Advia 1650-Chemistry System analyser (Siemens Healthcare Diagnostics, Eschborn, Germany). We measured fasting plasma glucose (FPG) on a Roche/Hitachi Cobas c 311 analyzer (Basel, Switzerland). Triglycerides and high-sensitivity C-reactive protein (CRP) with the Roche/Hitachi Cobas c311 analyser (Roche diagnostics, Mannheim, Germany), interleukin (IL)-6 with the Human IL-6 Quantikine HS ELISA (R&D Systems, Wiesbaden, Germany), IL-18 with the Human IL‐18 ELISA (Medical and Biological Laboratories, Nagoya, Japan), adiponectin with the Human Total Adiponectin/Acrp30 Quantikine ELISA kit (R&D Systems), chemerin with the Human Chemerin ELISA (BioVendor, Brno, Czech Republic), and leptin with leptin Quantikine ELISA (R&D System) as described [51, 52]. We considered SBP, DBP, triglyceride, HDL-c, FPG, CRP, IL‐6, IL‐18, adiponectin, chemerin, and leptin for the current study.

### Statistical analysis

#### Basic characteristics

Continuous and categorical variables are presented as median and interquartile range (IQR), and as counts (n) and percentages (%), respectively. Comparisons between sexes were performed using the Wilcoxon-Mann–Whitney test (continuous variables) and Chi square test (categorical variables).

#### Trajectory model building

BMI was calculated from body weight and height in each age year. We considered the latest measurement in an age year when multiple measurements existed. For trajectories, it is recommended that standard deviation scores (SDS) are modelled since the variance of weight increases rapidly with age in the first two decades of life [19]. Therefore, we calculated the BMI SDS with the LMS method based on age- and sex-specific median (M), coefficient of variation (S), and measure of skewness (L) values of the national German reference [53].

For each sex, we modelled trajectories of BMI SDS using a version of the LCGM that ensures that trajectory variation lies at the between-group level [54]. The procedure also assumes that attrition and group assignment are independent [55], and it automatically incorporates missing data under a missing at random assumption [56]. We performed the LCGM with the Statistical Analytical Software (SAS) function ‘proc traj’ [57]. We conducted sensitivity analysis to investigate whether participants who had complete BMI measurements differ from those who had one or more missing with respect to baseline determinants.

Without any a priori hypothesis, we investigated the possible number of trajectories and their shapes. We considered four shapes: cubic degree, quadratic degree, linear, and constant polynomials. We began with a single model consisting of one group with a cubic, and then increased the number of groups until the number of trajectories that best fit the data was identified using the log_e_Bayes factor (≈ 2(∆Bayesian information criterion)) of six and above, all trajectories sizes greater than 5%, and average trajectory posterior probability (APP) greater than 0.70 [56]. After identifying the number of trajectories, we proceeded to identify their shape. Starting with the first trajectory, we reduced the polynomial orders until the highest order term for all trajectories resulted in P ≤ 0.05. This resulted in a final trajectory model. This final trajectory model was eventually used to describe our data. Posterior trajectory membership probabilities were calculated based on model parameter estimates and the participant’s trajectory membership assignment was based their highest posterior trajectory probability. The trajectories were labelled according to World Health Organisation SDS cut-offs [58].

#### Baseline determinants of trajectories

By fitting multinomial logistic regression using SAS function ‘proc logistic’ and taking the largest trajectory as the reference group, we regressed BMI SDS trajectory on birth weight and length (continuous), MppBMI (continuous), GWG (continuous), breastfeeding duration (continuous). Other variables were dichotomised: birth order: into firstborn child (yes or no), maternal education (high = technical school/high school and low = secondary school/primary school), maternal employment (employed = full-time and part-time, and not employed = others), and smoking household (yes or no). We estimated the odds ratio (OR), the odds of each determinant in other trajectory groups as compared to the reference group, with a model comprising all these determinants (multivariable adjusted).

#### Handling of missing baseline determinants

Baseline determinants were birth weight and length, MppBMI, GWG, breastfeeding duration, birth order, maternal education, maternal employment, and smoking household. All variables except birth weight and length had missing values (range: four to 63% in males, and two to 53% in females). There were 58 and 56 missing data patterns in males and females, respectively. These patterns did not show any specific/obvious structure and were therefore considered arbitrary. Using SAS function ‘proc mi’, we created a single imputed dataset in one burn in iterations with the fully conditional method, linear regression for continuous variables (MppBMI, GWG, and breastfeeding duration), logistic regression for ordinal categorical variables (birth order and smoking household), and discriminant function for nominal categorical variables (maternal education and maternal employment).

#### Relevance of BMI SDS trajectory for cardiometabolic risk markers

An unbiased random forest regression (RFR) algorithm based on conditional inference trees using the tree building R function ‘cforest’ and the variable importance calculating R function ‘varimpAUC’ were used to build models for estimating the importance of BMI SDS trajectory relative to age-specific BMI SDS for each sex. We used all the 15 BMI SDS, along with BMI SDS as independent variables for each cardiometabolic risk marker and a pro-inflammatory score. The pro-inflammatory score was calculated as the average of internally standardized (z-) scores of CRP, IL-6, IL-18, chemerin, adiponectin, and leptin (CRP z-score + IL-6 z-score + IL-18 z-score + chemerin z-score + adiponectin z-score × (−1) + leptin z-score)/n, where n is the number of available proinflammatory marker per individual). We used 500 decision trees and four independent variables at each split in the RFR. We computed importance scores of all independent variables as the average of the area under the curve values. Due to the expected correlations between these independent variables, we estimated conditional importance scores. These analyses included participants with available cardiometabolic risk markers (triglycerides: 185 males and 184 females; HDL-c: 190 males and 185 females; SBP and DBP: 239 males and 228 females; FPG: 193 males and 188 females; CRP: 159 males and 154 females; IL-6: 154 males and 152 females; IL-18:156 males and 154 females; adiponectiin: 157 males and 153 females; chemerin: 159 males and 154 females, leptin: 158 males and 149 females; pro-inflammatory score: 154 males and 152 females). Despite RFR being non-parametric, it is advisable to adjust for non-normality and non-constant variation through transformation [59]. Thus, we transformed each cardiometabolic risk marker with the optimal exponent obtained from Box-Cox transformation using the SAS function ‘proc transreg’ in models without independent variables. We performed sensitivity analysis to investigate whether participants with no cardiometabolic risk marker, those with one to 10 cardiometabolic risk markers, and those with all 11 cardiometabolic risk markers differ with respect to baseline characteristics.

#### The association between BMI SDS trajectory and cardiometabolic risk markers

We investigated the association between BMI SDS trajectory and cardiometabolic risk markers by fitting generalized linear models using the SAS function ‘proc glimmix’, under a Gaussian distribution and associated link being the previously derived Box-Cox exponent. We adjusted for unequal variances across trajectories. The overall effect of trajectory as well as linear trend was assessed. Finally, we performed pairwise comparisons between trajectories, and adjusted the 95% confidence interval (CI) of their mean differences for multiple comparisons with the Tukey–Kramer method. We considered P ≤ 0.05 as significant. Covariates to be included in the models were baseline variables that are determinants of trajectories and that are also associated with cardiometabolic risk markers, as well as their product term with trajectories, if P ≤ 0.05. In addition, we investigated whether total energy intake, alcohol consumption, and smoking status in late adolescence–young adulthood mediate any observed difference between trajectories.

## Results

### Basic characteristics

The basic characteristics of the participants, 354 males and 335 females included in the present analysis are shown in Table 1. At birth, females were lighter and shorter than males. Females more frequently had mothers who were employed than males. There were no sex differences with respect to other baseline characteristics. In late adolescence–young adulthood, daily energy intake, SBP, DBP, and FPG were higher in males than females, while the converse was the case for HDL-c, CRP, adiponectin, chemerin, and leptin.

**Table 1:** Basic characteristics of the study participants, 354 males and 335 females

	n (males, females)	Male	Female	*P* value
Baseline (prenatal, birth, and early life) parameters				
Birth weight, g^a^	354, 335	3590 (610)	3380 (560)	< 0.01
Birth length, cm^a^	354, 335	52 (3)	51 (3)	< 0.01
Maternal prepregnancy BMI, kg/m^2a^	319, 304	23.2 (4.6)	22.7 (4.4)	0.13
Maternal gestational weight gain, kg^a^	341, 327	13 (5)	12 (5)	0.81
First-born child^b^	290, 274	161 (56)	159 (58)	0.45
Breastfeeding duration, weeks^a^	288, 277	26 (29)	28 (31)	0.36
Maternal education, high^b^	131, 160	119 (91)	143 (89)	0.36
Maternal empolyment, employed^b^	130, 159	27 (21)	59 (37)	< 0.01
Smoking household^b^	211, 204	51 (24)	52 (25)	0.36
Dietary intake and lifestyle in late adolescence-young adulthood (18–39 years)				
Daily total energy intake, kcal/day^a^	225, 217	2417.9 (584.3)	1816 (449)	< 0.01
Alcohol drinkers^b^	233, 221	164 (70)	160 (72)	0.64
Smokers^b^	223, 221	40 (18)	34 (15)	0.47
Cardiometabolic risk markers in late adolescence-young adulthood (18–39 years)				
Serum triglycerides, mg/dL^a^	185, 184	79 (50)	88 (49)	0.14
Serum HDL cholesterol, mg/dL^a^	190, 185	50 (15)	65 (18)	< 0.01
Systolic blood pressure, mmHg^a^	239, 228	118 (16)	110 (15)	< 0.01
Diastolic blood pressure, mmHg^a^	239, 228	74 (12)	70 (12)	< 0.01
Fasting plasma glucose, mg/dL^a^	193, 188	94 (11)	90 (9)	< 0.01
C-reactive protein, mg/dL^a^	159, 154	0.1 (0.1)	0.2 (0.3)	< 0.01
Interleukin-6, pg/mL^a^	154, 152	0.7 (0.6)	0.7 (0.6)	0.72
Interleukin-18, pg/mL^a^	156, 154	255.1 (101.8)	245.6 (96.7)	0.29
Adiponectin, ng/mL^a^	157, 153	6030.3 (5198.3)	8711.2 (5539.9)	< 0.01
Chemerin, ng/mL^a^	159, 154	140.9 (35.6)	166 (41.9)	< 0.01
Leptin, pg/mL^a^	158, 149	2335.4 (3441.2)	12666.5 (9517.4)	< 0.01

^a^ Median (interquartile range) and ^b^ n(%), n = count,  % = percentage, *BMI* body mass index. P-values of the difference between sexes were obtained from Wilcoxon-Mann–Whitney test for continuous variables, and Chi square test for categorical variables

Table 2 shows BMI development over the follow-up. As expected, BMI increases with age. The highest prevalence of overweight (including obesity) was about 10% in males (age 17) and 8% (age four) in females, and obesity alone was about 4% in males (age 18) and 3% (age four) in females. Notably, the BMI SDS shows that females generally had lower BMI SDS than males, particularly at ages 5, 6, 9, 10, 11, 17, and 18. These indicate an obvious sex differences and the need for sex-specific trajectories.

**Table 2:** Development of body mass index and body mass index standard deviation scores over the follow-up according to sex

Follow-up age (years)	n (males, females)	BMI, kg/m^2^, median (IQR)		% Overweight* (males, females)	% Obesity** (males, females)	BMI SDS, median (IQR)		P-value
		Males	Females			Males	Females	
4	137, 120	15.59 (1.58)	15.25 (1.70)	7.3, 8.3	1.5, 2.5	− 0.05 (1.32)	− 0.20 (1.33)	0.23
5	286, 267	15.52 (1.41)	15.21 (1.65)	6.6, 5.2	0.7, 0	0.08 (1.00)	− 0.15(1.21)	0.04
6	300, 287	15.48 (1.50)	15.29 (1.85)	6.3, 7.3	2.3, 0.7	0.00 (0.99)	− 0.13 (1.27)	0.04
7	311, 303	15.66 (1.75)	15.41 (2.07)	5.5, 7.9	1.9, 1.0	− 0.06 (1.03)	− 0.21 (1.24)	0.19
8	330, 313	15.91 (2.15)	15.78 (2.43)	5.8, 7.0	1.8, 1.0	− 0.13 (1.08)	− 0.20 (1.26)	0.13
9	350, 323	16.48 (2.51)	16.22 (2.60)	6.3, 5.0	0.6, 0.3	− 0.09 (1.10)	− 0.21 (1.21)	0.04
10	350, 323	17.12 (2.95)	16.61 (3.18)	6.0, 4.3	0.3, 0.3	− 0.07 (1.14)	− 0.28 (1.31)	0.01
11	345, 326	17.66 (3.30)	17.13 (3.46)	7.0, 3.4	0.6, 0	− 0.12 (1.17)	− 0.32 (1.29)	0.02
12	343, 323	18.36 (3.77)	18.1 (3.89)	6.1, 3.4	1.7, 0	− 0.14 (1.27)	− 0.23 (1.35)	0.15
13	340, 323	19.16 (3.78)	18.77 (3.76)	5.8, 4.6	1.5, 0	− 0.13 (1.27)	− 0.26 (1.29)	0.40
14	337, 316	19.76 (3.84)	19.64 (3.80)	6.2, 5.4	1.5, 0.3	− 0.17 (1.24)	− 0.21 (1.31)	0.39
15	335, 315	20.34 (4.25)	20.31 (3.74)	8.7, 5.1	1.2, 0	− 0.18 (1.40)	− 0.20 (1.29)	0.50
16	314, 289	21.07 (3.84)	20.89 (3.55)	9.6, 5.5	2.5, 0.3	− 0.10 (1.24)	− 0.16 (1.23)	0.14
17	287, 265	21.78 (4.37)	21.19 (3.66)	10.1, 4.9	2.4, 1.1	0.03 (1.38)	− 0.17 (1.27)	0.01
18	265, 256	22.21 (4.00)	21.47 (3.83)	9.1, 7.0	3.8, 1.2	0.13 (1.25)	− 0.10 (1.33)	< 0.01

*BMI* body mass index, *IQR* interquartile range, *SDS* standard deviation scores, n = count,  % = percentage. *BMI > 90th and **BMI > 97th age- and sex-specific percentile, according to the national German reference [53]. P-values of the difference between sexes were obtained from Wilcoxon-Mann–Whitney test

#### BMI SDS trajectory model development

There was a median of 14 (range: 5–15) BMI measurements. There were 67 and 68 missing BMI SDS patterns in males and females, respectively. These patterns have no specific/obvious structure and we therefore considered them arbitrary. Thus, parameter estimates of the trajectory models were unlikely to be biased. Sensitivity analysis also showed that participants who had complete BMI measurements were not different from those who had one or more missing measurement with respect to baseline determinants (Additional file 1: Table S1).

**Fig. 1:**
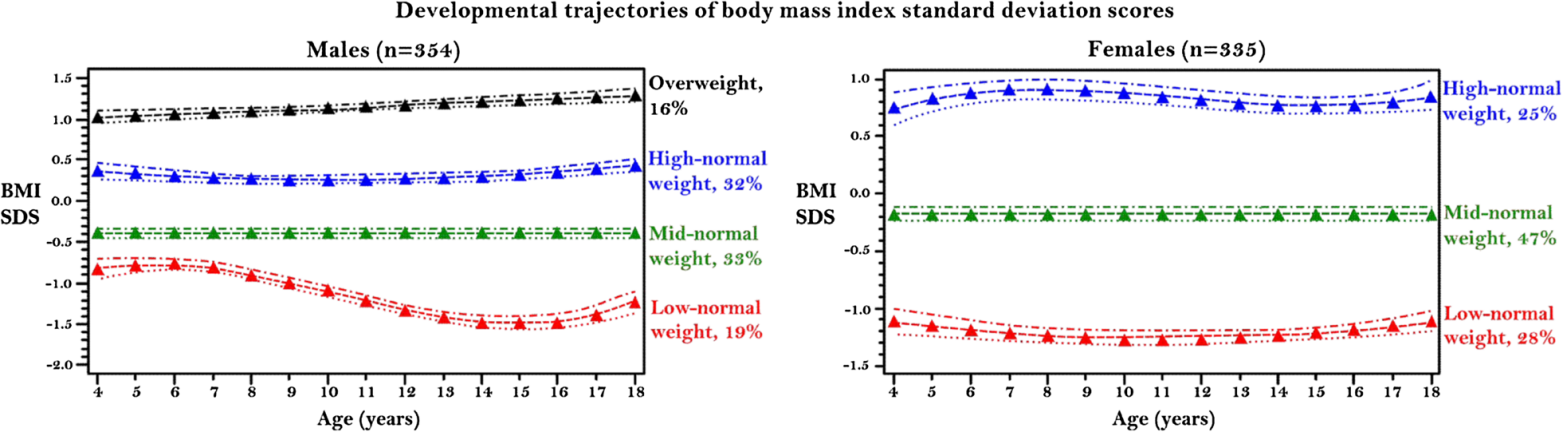
Developmental trajectories of body mass index standard deviation scores, and its upper and lower confidence intervals among males (left) and females (right) followed from ages 4 to 18. *BMI SDS* body mass index standard deviation scores

Based on the logeBayes factors, APP, and trajectory sizes, a four-trajectory group and a three-trajectory group was the most optimal for males and females, respectively. Figure 1 (left: males and right: females) shows the graph of the predicted BMI SDS for each trajectory group across the 15 years of analysis. In males, the first trajectory (red) consistently remained below other trajectories. The trajectory was S-shaped, with BMI SDS increasing from age 4 to 6, decreasing from age 6 to 15, and increasing from age 16 to 18. This group comprised 19% of the males. The second trajectory (green) comprised the largest (33%) proportion of males had relatively constant BMI SDS throughout the time of analysis, and the third trajectory (blue) accounting for 32% was U-shaped, with peaks at ages 4 and 18 years. These three trajectories were within the normal weight BMI SDS range (> − 2 to < + 1). Therefore, we named them ‘low-normal weight’, ‘mid-normal weight’, and ‘high-normal weight’ trajectories, respectively. The fourth trajectory (black) that accounted for 16% of the males had a BMI SDS starting at overweight BMI SDS of 1.00, and BMI continues to increase throughout follow-up. This group was named as the ‘overweight’ trajectory.

In females, the first trajectory (red) accounting for 28% was U-shaped, with peaks at ages 4 and 18 years. The second (green) which is composed of the largest (47%) proportion of females had a relatively constant BMI SDS throughout follow-up. The third (black) accounting for 25% of the population had S-shaped trajectory, with BMI SDS increasing from age 4 to 8, decreasing from age 8 to 14, and increasing from age 15 to 18. All three trajectories were within the normal weight range, and we named them ‘low-normal weight’, ‘mid-normal weight’, and ‘high-normal weight’ trajectories, respectively.

All trajectories were distinct without overlap at any point. Their parameter estimates, associated standard errors and P-values are presented in Additional file 2: Table S2.

#### Baseline determinants of BMI SDS trajectory

**Fig. 2:**
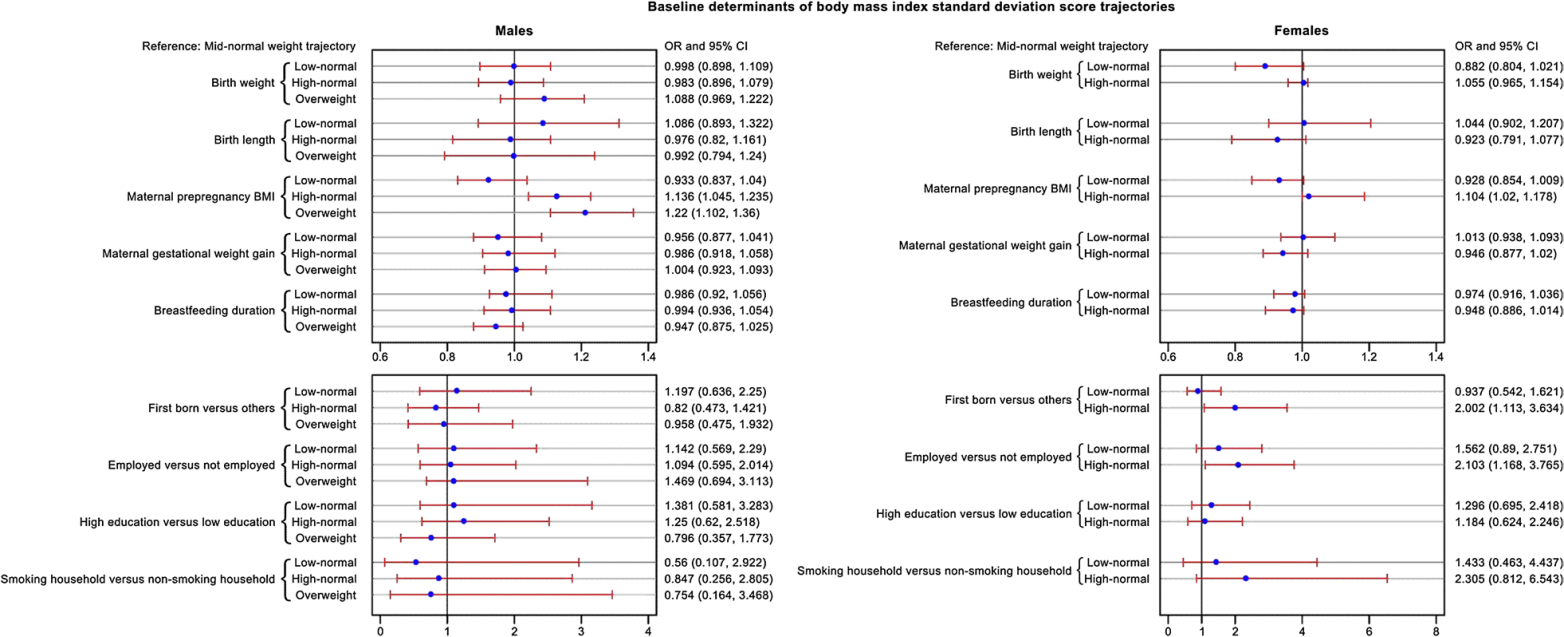
Forest plot of the adjusted odds ratios of the baseline determinants of body mass index standard deviation scores trajectories among males (left) and females (right). The odds ratio plot showing the point estimate of the odds ratio, and surrounding confidence intervals and a reference line at 1 (tests of significance). The reference group is ‘mid-normal weight’ trajectory. *OR*  odds ratio, *CI* confidence interval, *BMI* body mass index. OR per 100 g increase in birth weight, per 1 cm increase in birth length, per 1 kg/m^2^ increase in maternal prepregnancy BMI, per 1 kg increase in gestational weight gain, and per 1 month increase in breastfeeding duration

As shown in Fig. 2 (left: males and right: females), sons of mothers with a high BMI were more likely to follow the ‘high-normal weight’ (adjusted OR = 1.14; 95% CI 1.05–1.24) and ‘overweight’ (adjusted OR = 1.22; 95% CI 1.10–1.36) trajectories as compared to those whose mothers had a lower BMI. Similarly, daughters of mothers with a high BMI were more likely to follow the ‘high-normal weight’ trajectory (adjusted OR = 1.10; 95% CI 1.02–1.18). Compared to females who followed the ‘mid-normal weight’ trajectory, females who followed the ‘high-normal weight’ trajectory were more likely to be first-born (adjusted OR = 2.00; 95% CI 1.10–3.63) and their mothers were more likely to be employed (adjusted OR = 2.10; 95% CI 1.17–3.77).

#### Relevance of BMI SDS trajectory for cardiometabolic risk markers

**Fig. 3:**
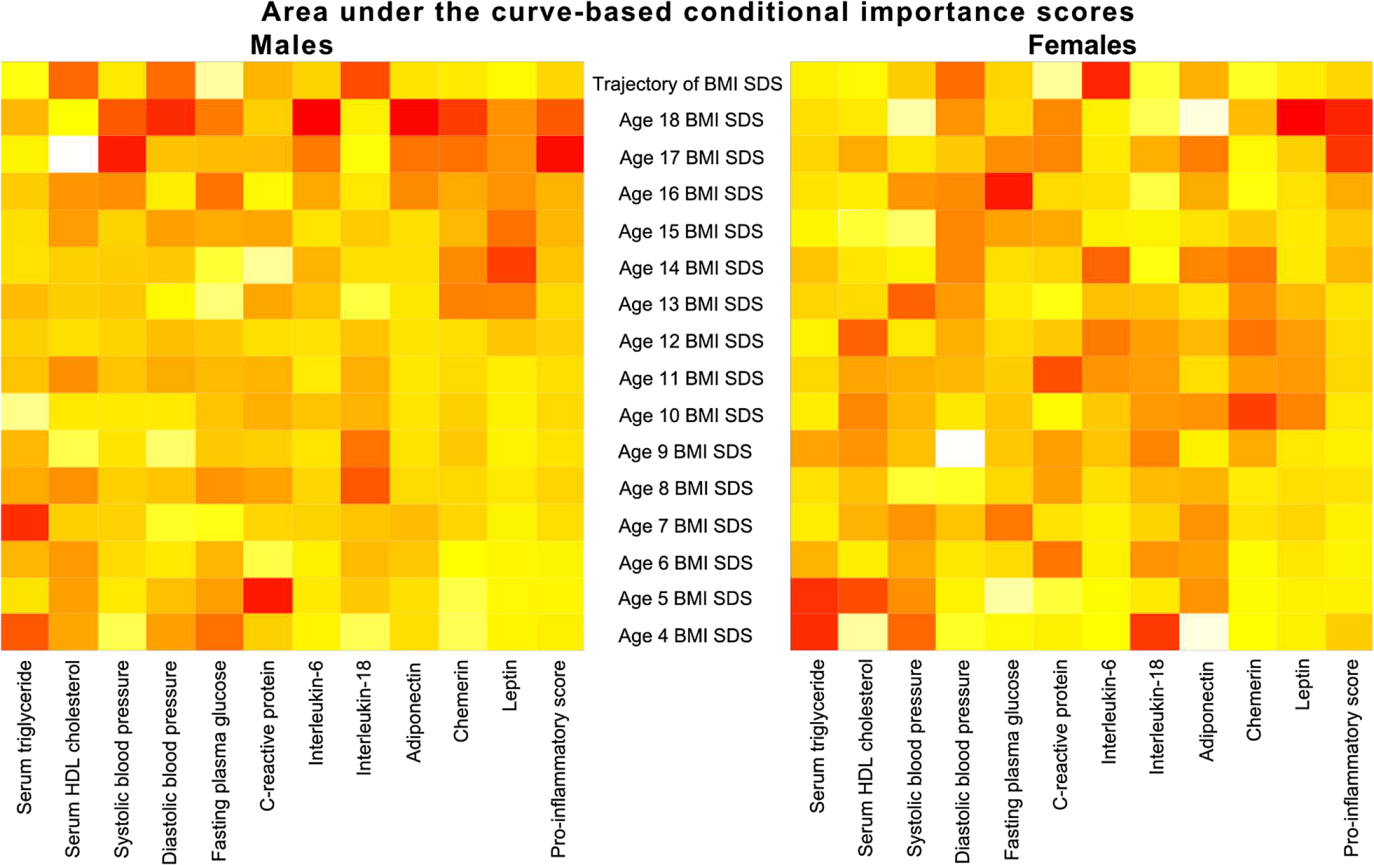
Heat maps showing the area under the curve-based conditional importance scores determined by random forest for males (left) and females (right) for BMI SDS trajectory relative to BMI SDS at specific ages for cardiometabolic risk markers in late adolescence–young adulthood. The colour order of magnitude of the importance scores is from yellow→orange→red (highest values are in red and lowest values are in yellow). *BMI SDS* body mass index standard deviation scores. Analysis performed on transformed cardiometabolic risk markers. Transformation (exponents) were: In males, triglycerides, IL-6 and leptin were transformed by natural logarithm; HDL-c by square root; pro-inflammatory score by cube root; adiponectin by fourth root; SDP, CRP, and chemerin by inverse of fourth root; DBP and FPG by cube of fourth root; and IL-18 by inverse of the cube of fourth root. In females, triglycerides, CRP, adiponectin, and leptin were transformed by natural logarithm; HDL-c and chemerin by square root, pro-inflammatory score by cube root; DBP by cube of square root; and SBP, FPG, IL-6, IL-18 by inverse of fourth root

The relevance (importance) of BMI predictors of each cardiometabolic risk marker are presented as heat maps in Fig. 3 (left: males and right: females). After conditioning on all age-specific BMI SDS, BMI trajectory was strongly associated with HDL-c and IL-18 in males and with DBP and IL-6 in females. In males, BMI SDS at age four was strongly associated with FPG, age five with CRP, age seven with triglycerides, age 14 with leptin, age 17 with leptin, SBP and pro-inflammatory score, and age18 with DBP, IL-6, adiponectin, and chemerin. In females, BMI SDS at age four was strongly associated with triglyceride and IL-18, age five with HDL-c, age 10 with chemerin, age 11 with CRP, age 13 with SBP, age 16 with FPG, age 17 with adiponectin, and age18 with leptin and pro-inflammatory score.

Sensitivity analysis showed that males without cardiometabolic risk markers were breastfed longer, had the lowest proportion of mothers with higher education, and the highest proportion of employed mothers. In addition, females without cardiometabolic risk markers were heavier and taller (Additional file 3: Table S3).

#### The association between BMI SDS trajectory and cardiometabolic risk markers

In males, trajectory had no significant overall association with HDL-c (P = 0.43) but with IL-18 (P = 0.04) whereas in females it was neither associated with DBP (P = 0.27) nor with IL-6 (P = 0.11). Moreover, no linear trend was observed for the four cardiometabolic risk markers (HDL-c: P_trend_ = 0.38, IL-18: P_trend_ = 0.12, DBP: P_trend_ = 0.19, and IL-6: P_trend_ = 0.15).

**Fig. 4:**
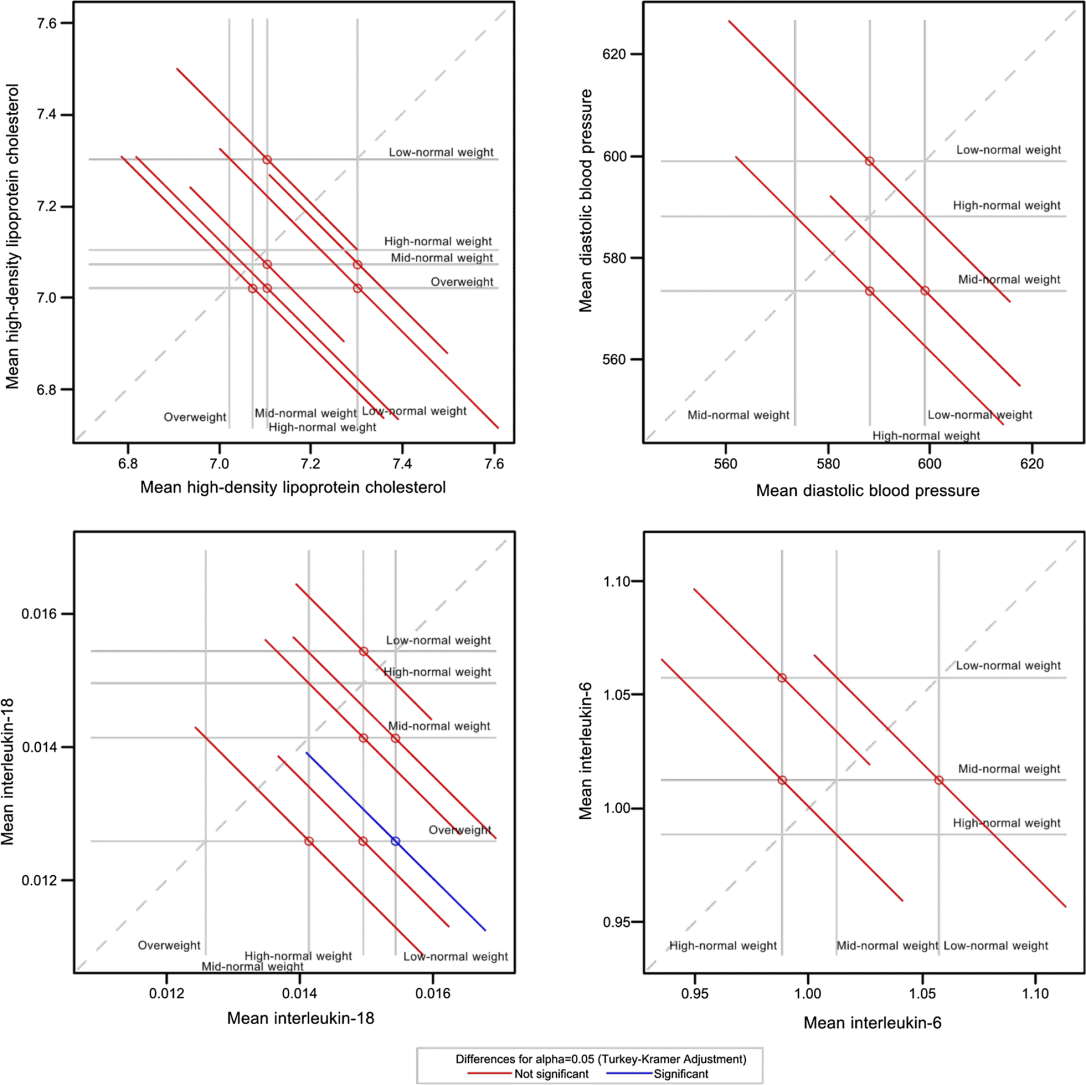
Diffograms showing pairwise comparisons of body mass index standard deviation score trajectories for high-density lipoprotein cholesterol and interleukin-18 in males (left), and for diastolic blood pressure and interleukin-6 in females (right) in late adolescence–young adulthood. The dashed diagonal upward sloping reference line depicts equality. The horizontal and vertical lines emanating from the axes indicate the location of the means of the four BMI trajectory groups. There are four vertical and four horizontal reference lines, and six pairwise comparisons of the means in males. There are three vertical and three horizontal reference lines, and three pairwise comparisons of the means in females. Each solid circle at the point of intersection of the horizontal and vertical lines shows the location of mean of two BMI trajectories and the associated diagonal line segment represents the Tukey–Kramer adjusted 95% confidence interval for the difference between the means. A confidence interval that intersect (red colour) the dashed diagonal line indicates that those two means are not statistically different and significant (blue colour) if otherwise. Mean displayed on the diffogram are on the exponents of cardiometabolic risk markers. Exponents: square root for HDL-c, inverse of the cube of fourth root for IL-18, cube of square root for DBP, and inverse of fourth root for IL-6

There was no significant difference between all pairs of trajectories for HDL-c (Fig. 4, upper left) as well as for DBP and IL-6 (Fig. 4, upper right and bottom right). However, the mean IL-18 in the ‘overweight’ trajectory was significantly higher than that in the ‘low-normal weight’ trajectory (Fig. 4, bottom left), (mean difference = 2.85 × 10^−3^ pg/mL (0.14 × 10^−3^–5.55 × 10^−3^) transformed scale; 81.31 pg/mL (33.62–196.24) original scale. The baseline determinants of trajectories: MppBMI, maternal employment, and first-born status were not associated with any of the four cardiometabolic risk markers. Their product terms with trajectory were also not significant. Therefore, adjustment for these covariates was deemed unnecessary. Furthermore, total energy intake and smoking was neither associated with trajectory nor with IL-18, however, alcohol consumption was associated with BMI trajectory, but not with IL-18. Thus, the difference between IL-18 in ‘overweight’ and ‘low-normal weight’ trajectories is unlikely to be explained by baseline factors or dietary intake and lifestyle factors in late adolescence–young adulthood.

## Discussion

Using annual BMI measurements from childhood into late adolescence (ages 4 to18) in a German cohort, we identified four distinct BMI SDS trajectories in males–three in which BMI SDS remains within a normal weight range (84%), and one ‘overweight’ trajectory (16%), and in females–three trajectories, all within normal weight range. The normal weight trajectories were named ‘low-normal weight’, ‘mid-normal weight’, and ‘high-normal weight’ based on the intensity of BMI SDS. The MppBMI was a determinant of the ‘overweight’ trajectory in males, and a common determinant of ‘high-normal weight’ trajectory in both sexes. Additionally, employed mother and first-born status were determinants of ‘high-normal’ trajectories only in females. Furthermore, BMI SDS trajectory was highly associated with HDL-c and IL-18 in males, and DBP and IL-6 in females. Importantly, circulating levels of IL-18 were elevated in males who followed the ‘overweight’ trajectory when compared to males who followed the ‘low-normal weight’ trajectory.

### BMI SDS trajectory model development

Our findings of sex-specific trajectories, both in terms of numbers and shapes is consistent with reports of sex differences in BMI at specific periods in childhood and adolescence [60], and particularly in the trajectories of BMI [15, 20]. Thus, our study extends the previous literature on male–female differences. Possible explanations for this finding include differences in sex hormones [42], and differential exposure and vulnerability to obesogenic environments in males and females [60]. The fact that our trajectories do not overlap is in accordance with a recent analysis showing that adiposity status in German adolescents was already set in early childhood [7], and that children and adolescents with increasing weight gain usually continue on this path [4–8]. Therefore, it is crucial that individuals who are overweight in childhood and are persistently gaining weight like those captured by our ‘overweight’ trajectory should be monitored, as these individuals are prone to develop obesity later on. The present study also extends the results from other studies using LCGM [9, 11, 12, 15, 17, 19, 20], which have shown the existence of three to four adiposity trajectories from childhood into late adolescence.

### Baseline determinants of BMI SDS trajectory

Our study demonstrated that MppBMI is a crucial determinant of high BMI trajectories being a common determinant in both sexes. There is compelling evidence that high MppBMI is associated with high adiposity trajectories [9, 12, 15, 16, 61]. Genetic factors, early programming, and obesogenic environment are possible explanations [60]. Indeed, this somewhat modifiable early-life risk factor warrants attention. It is imperative to advise women, particularly those who intend to become pregnant of the need to have and maintain a normal BMI.

In addition, we observed employed mothers and first-born status to increase the risk of females following the ‘high-normal’ trajectory. These factors have not been previously linked to BMI trajectory. This maternal employment patterning of BMI trajectory in females is in line with evidence of greater parental influence on increased adiposity in females [60], and socioeconomic differences in adiposity in developed countries that is more obvious among females than males [62]. This finding is also consistent with a positive association between maternal employment and offspring adiposity at specific phase of life among well-educated populations [63, 64]. The interrelationship of maternal employment, higher family income, and children access to healthier lifestyles seems obvious at first glance. However, maternal employment also implies that an important role model in a child’s adoption of healthy behaviours is available for a lesser amount of time and that childcare is shifted to other caring parent, informal caregivers or formal providers. In agreement with our finding, others have reported association between first‐born status and overweight in women [65]. The underlying mechanisms for this finding is still unknown, however in utero triggering events have been speculated [65].

### The relevance of BMI SDS trajectory for cardiometabolic risk markers

BMI trajectory was strongly associated with HDL-c and IL-18 in males, and DBP and IL-6 in females. This shows that BMI trajectory confers additional cardiometabolic risk beyond age-specific BMI. Interestingly, BMI trajectory was associated with cytokine levels in both sexes. This suggests a strong impact of BMI trajectory on this group of proinflammatory markers. Further, the sex-specific trajectory–cardiometabolic risk marker association is noteworthy. This suggests that sexual dimorphism associated with some long-term health consequences is related to events during childhood and adolescence. Although a sex-specific trajectory–cardiometabolic risk markers association has not been previously reported, sex disparity in cross-sectional relation between BMI and BP [27, 38], BMI and HDL-c [37, 45], BMI and IL-6 [33, 34], and BMI and IL-18 [33] in adolescents and young adults is documented.

The link between BMI trajectory and IL-18, and the fact the ‘overweight’ and ‘low-normal weight’ trajectories in males were distinguishable is intriguing, considering the low prevalence of overweight among our study population when compared to the general German population [66]. IL-18 may represent a cardiometabolic signature of ‘overweight’ trajectory in young adults. To the best of our knowledge, differences in late adolescence–young adulthood IL-18 with respect to BMI trajectories from childhood into late adolescence have not been reported. Nonetheless, there is evidence of a cross-sectional relationship between BMI and late adolescence–young adulthood IL-18 [27, 32]. This suggests that our finding is unlikely to be spurious. Therefore, IL-18 might be a useful marker of long-term overweight. In fact, it could be clinically relevant considering the substantial difference between the overweight and ‘low-normal weight’ trajectories. Early identification and intervention for late adolescents–young adults who are overweight and with a history of persistent weight gain is imperative. IL-18 is a unique proinflammatory cytokine, a member of the IL-1 family of cytokines that is produced by the adipocytes [67], and other cell types such as macrophages, endothelial cells, vascular smooth muscle cells, dendritic cells and Kupffer cells [68]. Human studies have consistently demonstrated that higher levels of IL-18 is an independent risk factor for incident type 2 diabetes mellitus [68, 69], and cardiovascular events [68, 70–73]. This indicates that elevated IL-18 may put these young males following the ‘overweight’ trajectory at risk of future cardiometabolic diseases.

The current study cannot corroborate previous findings of lower levels of HDL-c in individuals following the ‘higher BMI growth’ as compared to those following the ‘average BMI growth’ [16], and females in the ‘upward percentile crossing’ group having higher DBP than other groups [12]. These divergent findings could be attributable to differences in statistical approaches for estimating trajectories, differences in modelled BMI metrics, absence of a comparable ‘upward percentile crossing’ group among our females, and our study may be underpowered to detect minor between-trajectory differences in HDL-c and DBP. Furthermore, circulating retinol-binding protein-4 [74, 75] and plasminogen activator inhibitor-1 [76] correlate with BMI and some cardiometabolic risk markers in children and adolescents, and measures of HDL-c function as compared to HDL-c levels provide a better assessment of cardiovascular risk [77]. Therefore, these adipokines and measures of HDL-c function should be considered in future studies.

The strengths of this study are that we objectively measured weight and weight at all ages; as such, we can exclude misreporting. Moreover, the annual measurements over this relatively long period of follow-up suggests that the yielded trajectories are accurate. Our time sequence of trajectory and cardiometabolic risk markers also confirms cause-and-effect relationships of BMI trajectory and IL-18. However, we acknowledge several study limitations. First, our participants are mainly Caucasians (Germans), residing in Dortmund and surrounding regions, and most are children of well-educated women, thus our findings are of limited generalizability. We investigated all determinants at once around birth; however, family and socioeconomic characteristics might change over the life course. Participants who were not included in the analyses for the cardiometabolic risk markers may have had different associations between BMI trajectories and IL-18. However, the fact that the baseline factors that differentiate these groups were not associated with trajectories suggest these findings are unlikely to be biased. Importantly, other unmeasured factors such as the gut microbiota may have influenced the present findings. Finally, cardiometabolic risk markers were collected once at the end of the follow-up. Assessment of cardiometabolic risk markers at few time-points that coincides with BMI measurements and identifying latent clusters of individuals with joint evolvement of BMI and these markers might provide a deeper understanding of association between developmental trajectories of BMI and cardiometabolic risk markers.

## Conclusion

In a group of individuals with low prevalence of overweight, we observed distinct sex-specific trajectories of BMI, MppBMI as a common determinant of high BMI trajectories and IL-18 distinguished between males who followed the ‘overweight’ and ‘low-normal’ trajectories. Women maintaining normal pregravid weight might prevent their children following an ‘overweight’ trajectory. IL-18 may represent a cardiometabolic signature of ‘overweight’ trajectory in young adults. Given the increasing prevalence of overweight among German young adults, there is a need for confirmation of these findings in other cohorts with a larger study sample.

## Additional files

**Additional file 1: Table S1.** Comparison of study population with complete body mass index measurements and those who had one or more missing measurements according to baseline characteristics. *Median (interquartile range) and ^#^ n (%), n = count, % = percentage. P-values for difference between groups were obtained from Wilcoxon-Mann-Whitney test for continuous variables and chi-square test for categorical variables.

**Additional file 2: Table S2.** The parameter estimates, standard errors, P-values and average posterior probabilities of the four- and three-trajectory model in males and females. n = counts, BIC = Bayesian information criterion.

**Additional file 3: Table S3.** Comparison of study sample with no cardiometabolic risk marker, those with one to 10 markers, and those with complete markers according to baseline characteristics. *Median (interquartile range) and ^#^ n (%), n = count, % = percentage. P-values for difference among groups were obtained from Kruskal Wallis test for continuous variables and chi-square test for categorical variables.

## Abbreviations

APP: average trajectory posterior probability
BMI: body mass index
BP: blood pressure
CI: confidence interval
DBP: diastolic blood pressure
DONALD: DOrtmund Nutritional and Anthropometric Longitudinally Designed
FPG: fasting plasma glucose
GWG: gestational weight gain
HDL-c: high-density lipoprotein-cholesterol
IQR: interquartile range
IL: interleukin
LCGM: latent (class) growth models
L: measure of skewness
M: median
MppBMI: maternal prepregnancy BMI
OR: odds ratio
RFR: random forest regression
S: coefficient of variation
SAS: Statistical Analytical Software
SBP: systolic blood pressure
SD: standard deviation
SDS: standard deviation scores
